# Characterization of insect-specific Culex flavivirus (*Flaviviridae*) nucleotide sequences in mosquitoes from urban parks in São Paulo, Brazil

**DOI:** 10.1590/0037-8682-0067-2022

**Published:** 2022-09-26

**Authors:** Thaís de Moura Coletti, Camila Malta Romano, Paulo Roberto Urbinatti, Renildo Souza Teixeira, Leila Weiss de Almeida Pedrosa, Marcello Schiavo Nardi, Delsio Natal, Antônio Charlys da Costa, Jaqueline Goes de Jesus, Ingra Morales Claro, Ester Cerdeira Sabino, Steven S. Witkin, Mauro Toledo Marrelli, Licia Natal Fernandes

**Affiliations:** 1 Universidade de São Paulo, Faculdade de Medicina, Instituto de Medicina Tropical, Laboratório de Investigação Médica-49, São Paulo, SP, Brasil.; 2 Universidade de São Paulo, Faculdade de Medicina, Instituto de Medicina Tropical, Laboratório de Investigação Médica-46, São Paulo, SP, Brasil.; 3 Universidade de São Paulo, Faculdade de Medicina, Hospital das Clínicas, São Paulo, SP, Brasil.; 4 Universidade de São Paulo, Faculdade de Saúde Pública, Departamento de Epidemiologia, São Paulo, SP, Brasil.; 5 Prefeitura de São Paulo, Centro de Controle de Zoonoses, Laboratório de Fauna Sinantrópica, São Paulo, SP, Brasil.; 6 Secretaria do Verde e Meio Ambiente, Divisão da Fauna Silvestre, Coordenadoria de Gestão de Parques e Biodiversidade, São Paulo, SP, Brasil.; 7 Universidade de São Paulo, Faculdade de Medicina, Instituto de Medicina Tropical, São Paulo, SP, Brasil.; 8 Weill Cornell Medicine, Department of Obstetrics and Gynecology, New York, NY, USA.

**Keywords:** Culex flavivirus, Insect-specific flavivirus, Mosquito, Urban park

## Abstract

**Background:**

Despite their worldwide occurrence, the distribution and role of insect-specific flaviviruses (ISFs) remain unclear.

**Methods:**

We evaluated the presence of ISFs in mosquitoes collected in São Paulo, Brazil, using reverse transcription and semi-nested polymerase chain reaction (PCR). Some of the positive samples were subjected to nanopore sequencing.

**Results:**

Twelve mosquito pools (2.8%) tested positive for flavivirus infection. Nanopore sequencing was successfully performed on six samples. Phylogenetic analysis grouped these sequences into genotype 2 of Culex flavivirus (CxFV).

**Conclusions:**

The identification of CxFV genotype 2 at new locations in São Paulo highlights the importance of understanding the role of ISFs in mosquito vector competence.

In recent decades, PCR assays using degenerate primers targeting various flaviviruses have been increasingly used to monitor pathogenic flaviviruses and have contributed to the discovery of many insect-specific flaviviruses (ISFs)^1^ ^-^ ^3^ that replicate only in insects^4^ ^,^ ^5^. Several ISFs have been described worldwide, including Culex flavivirus (CxFV)^4^ and others^5^.

CxFV is the most frequently identified ISF in Brazil. It was detected in mosquitoes collected in green areas in the cities of São Paulo-SP^2^, São José do Rio Preto-SP^3^, Macapá-AP^6^, Cuiabá-MT^7^, Vitória- ES, Curitiba-PR and Ribeirão Claro/Carlópolis-PR^8^. In addition, Aedes flavivirus (AEFV) was detected in the city of São Paulo^2^ and in Curitiba^8^, and cell- fusing agent viruses (CFAV) was identified in Macapá^6^. Finally, a novel ISF, Sabethes flavivirus (SbFV), was discovered in Ribeirão Claro/Carlópolis^8^.

To date, knowledge about ISFs is incomplete. However, given the occurrence of large epidemics of flaviviruses in Brazil that infect humans, including dengue virus (DENV), zika virus (ZIKV), and yellow fever virus (YFV)^9^ ^,^ ^10^, it is crucial to understand the potential involvement of ISFs in the dynamics of these infections. In addition, there is a lack of information on the occurrence, frequency, and distribution of ISFs, as well as on their host range. Therefore, the present study aimed to identify and characterize ISFs in mosquitoes collected from parks in urban areas of São Paulo.

Mosquitoes (Diptera: Culicidae) were collected in urban parks in São Paulo, the state of São Paulo, southeastern Brazil. São Paulo has over 12.3 million people and is located in a highly urbanized area, where parks are among the last places where biodiversity is protected and conserved.

Three parks were selected for this study: Burle Marx, Piqueri, and Previdência. Their areas and geographic coordinates are respectively: 480,000m^2^/23°37'56 "S 46°43'17" W, 98,000m2/23°31'39.98 "S 46°34'24.88" W, and 44,323m^2^/23°34'50" S 46°43'36 "W. The vegetation in these parks consists mainly of remnants of the native Atlantic Forest, together with artificially planted trees (mainly eucalypts), gardens, and grassy areas. Among the various animal species that inhabit these parks, the most notable is a wide variety of birds. These parks are widely used for sports and recreational activities. At the time of the study, there was no evidence that flaviviruses were circulating in the neighborhoods where the parks were located.

Mosquito samples were collected from each park monthly from August 2012 to July 2013. A total of 36 collections, 12 from each park, were performed. As previously described, mosquito collections were conducted during the day and over three hours from dusk, as described previously^2^. Mosquitoes were transported to the laboratory on dry ice and stored at -80◦C until use. Morphological identification was performed at approximately -10°C on a specially designed cooling table using a stereomicroscope and Forattini's dichotomous key^11^. Up to ten non-engorged females were pooled according to their taxonomic category, location, and date of collection. After identification, mosquitoes were stored in a freezer at -80 °C. Many mosquito pools were found in the collections at Piqueri Park, so they were randomly selected, and only 20% of them were analyzed.

Non-engorged female mosquito pools were thawed and homogenized in 1 ml Gibco® Hank’s Balanced Salt Solution using a pellet pestle motor (Sigma-Aldrich). Microtubes (1.5 ml) containing the samples were centrifuged (12,000 min/4 °C), and the supernatant (400 μl) was subjected to the Specific B/Lysis off-board protocol of NucliSENS® easyMag® (BioMérieux) according to the manufacturer's instructions. Nucleic acids were eluted in 80µl elution buffer and stored at -80ºC.

Reverse transcription was performed using the GoScript^TM^ Reverse Transcriptase kit (Promega) and random primers (Invitrogen). A semi-nested PCR was conducted according to the protocols described by Cook et al.^1^, with small modifications described by Fernandes et al.^2^. A final fragment of 200 bp of the flavivirus NS5 gene was obtained and resolved by electrophoresis on an agarose gel. Complementary DNA from the supernatants of DENV- and YFV-infected cell cultures were used as a positive control in the PCR. Water was used as a negative control.

Following PCR, the products were purified (GFXTM PCR DNA and Gel Band Purification Kit, GE Healthcare) and sequenced using a Big Dye Terminator^TM^ v3.1 Cycle Sequencing Ready Reaction - ABI Prism® (Applied Biosystems) on an Applied Biosystems 3100 Genetic Analyzer. Primers used were those from the second round of the semi-nested PCR. Nucleotide sequences were submitted to BLAST (Basic Local Alignment Search Tool (BLAST)) to detect similarities at the nucleotide level with sequences from GenBank.

Samples with positive results in the semi-nested PCR previously described were subjected to a PCR for the amplification of a 982 bp fragment of the NS5 gene of flavivirus, according to Fulop et al.^12^, to obtain a longer fragment for phylogenetic analysis. Subsequently, 2% E-gel™ EX agarose gels were used to visualize the fragments. Next, the expected fragments were sequenced using the step-by-step MinION sequencing protocol - 1D native barcoding genomic DNA (with SQK-LSK109)^13^. Finally, the generated data were sent for phylogenetic analysis.

A maximum likelihood phylogenetic tree was inferred using the PhyML program with the previously defined best-fit TrNef G + IG+I nucleotide substitution model. The Nearest-Neighbor-Interchange heuristic method was applied to search the optimal/sub-optimal tree. The support of the key nodes was obtained by bootstrapping with 1,000 replicates. Finally, the genetic distances were estimated using Geneious Prime 2020 (https://www.geneious.com).

A total of 3,043 Culicidae female mosquitoes divided into 415 pools were screened by semi-nested PCR for the 200 bp fragment of the NS5 gene of flavivirus. Table 1 shows the number of Culicidae females (and pools) analyzed according to the taxonomic category and place of collection. Regarding the taxonomic classification of the mosquitoes, 1,507 specimens (199 pools) consisted of *Culex* and 1,479 specimens (189 pools) of *Aedes*. Mosquitoes from the other four genera (*Anopheles*, *Coquillettidia*, *Limatus*, and *Psorophora*) were less abundant and together comprised only 57 specimens (27 pools).

**TABLE 1:** Number of Culicidae females analyzed according to taxonomic category and place of collection.

Taxonomic category	Place of Collection			
	Burle Marx	Previdência	Piqueri	Total
*Aedes aegypti*	-	**2** (2)	**2** (2)	**4** (4)
*Aedes albopictus*	**21** (6)	**39** (10)	**1** (1)	**61** (17)
*Aedes fluviatilis*	**96** (16)	**169** (23)	**165** (17)	**430** (56)
*Aedes scapularis*	**391** (44)	**434** (50)	**159** (18)	**984** (112)
*Anopheles* sp*.*	**1** (1)	**1** (1)	-	**2** (2)
*Anopheles evansae*	**1** (1)	-	-	**1** (1)
*Anopheles strodei*	**26** (6)	-	-	**26** (6)
*Coquillettidia juxtamansonia*	**1** (1)	-	-	**1** (1)
*Culex chidesteri*	**12** (6)	**1** (1)	**1** (1)	**14** (8)
*Culex (Culex)* sp.	**157** (22)	**149** (21)	**210** (21)	**516** (64)
*Culex (Mel.)* Section *Melanoconium*	**20** (7)	-	-	**20** (7)
*Culex nigripalpus*	**348** (42)	**231** (30)	**344** (37)	**923** (109)
*Culex quinquefasciatus*	**7** (6)	**9** (2)	**18** (3)	**34** (11)
*Limatus durhami*	**16** (10)	**8** (4)	-	**24** (14)
*Psorophora ferox*	**2** (2)	-	**1** (1)	**3** (3)
**Total**	**1,099** (170)	**1,043** (144)	**901** (101)	3,043 (415)

In bold = number of mosquito specimens, in parentheses = number of mosquito pools.

A total of 12 pools (2.8%) were positive for flavivirus; in 11 of them, the 200 bp fragment was successfully sequenced. Eight of the positive pools were detected in mosquitoes from Burle Marx Park and four in mosquitoes from Piqueri Park. All samples from the Previdência Park were flavivirus-negative. MinION Nanopore Sequencing of the 982 bp fragment of the NS5 gene of flavivirus was successfully performed in six of the positive samples. Information about the positive samples, including the nucleotide sequence similarity obtained after BLAST analysis, is shown in Table 2. The 200 bp sequences could not be submitted to GenBank because of their short size.

**TABLE 2:** Characteristics of the pools of Culicidae females in which Culex flavivirus sequences were detected: mosquito taxonomic category, number of specimens per pool, collection place (park), collection date, and nucleotide sequence similarity after BLAST analysis of the fragments obtained.

Pool code	Taxonomic category	Number of specimens per pool	Park	Collection Date	Nucleotide sequence similarity	
					200nt	982nt
SP BR 1	*Culex quinquefasciatus*	1	Burle Marx	10/9/2012	CxFV	-
SP BR 2	*Culex quinquefasciatus*	10	Piqueri	17/9/2012	CxFV	-
SP BR 3	*Culex* (*Culex*) sp.	10	Piqueri	17/9/2012	CxFV	CxFV
SP BR 4	*Culex* (*Culex*) sp.	10	Burle Marx	8/10/2012	CxFV	-
SP BR 5	*Culex quinquefasciatus*	1	Burle Marx	12/11/2012	CxFV	-
SP BR 6	*Culex* (*Culex*) sp.	5	Burle Marx	12/11/2012	CxFV	CxFV
SP BR 7	*Culex* (*Culex*) sp.	6	Burle Marx	6/12/2012	CxFV	CxFV
SP BR 8	*Culex* (*Culex*) sp.	7	Burle Marx	21/1/2013	CxFV	-
SP BR 9	*Culex* (*Culex*) sp.	12	Piqueri	21/2/2013	CxFV	CxFV
SP BR 10	*Culex nigripalpus*	3	Burle Marx	11/3/2013	CxFV	-
SP BR 11*	*Culex* (*Culex*) sp.	6	Burle Marx	11/03/2013	-	CxFV
SP BR 12	*Culex* (*Culex*) sp.	10	Piqueri	18/3/2013	CxFV	CxFV

*This sample was positive in the semi-nested PCR of the NS5 gene of flavivirus*,* although the 200 bp fragment obtained could not be successfully sequenced.

Phylogenetic analysis was performed using the 982 bp sequence of flavivirus obtained in our study and CxFV reference sequences retrieved from GenBank. A sequence of CFAV was used as an outgroup. In the phylogenetic tree (Figure 1), the sequences from the present study were grouped with sequences of CxFV previously detected in the Caribbean, Latin America, and Africa. None of the present sequences were grouped with CxFV reference sequences previously detected in the USA or Asia. A distance matrix with the genetic distances among the sequences from this group was constructed (data not shown).

**FIGURE 1:**
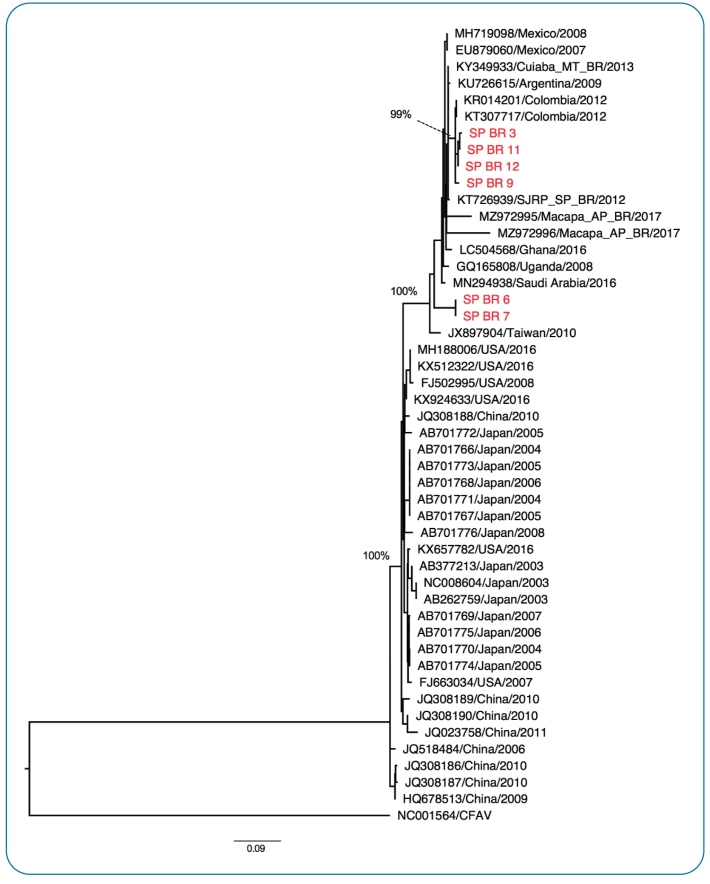
Maximum likelihood phylogenetic tree of the 982 bp fragments of the NS5 gene of flavivirus. In red, sequences generated in the present study (see additional information in Table 2). In black, CxFV reference sequences retrieved from GenBank (represented with their GenBank accession numbers, place, and date of sample collection). A reference sequence from CFAV was used as an outgroup.

CxFV was the only ISF identified in mosquitoes collected from urban parks in the city of São Paulo. The present findings corroborated a prior study on the occurrence of CxFV in the southern and eastern regions of this city^2^.

This ISF is commonly found in mosquitoes of the *Culex* genus and has been detected in multiple countries worldwide^5^. In Brazil, CxFV has been previously detected in mosquitoes collected in the Southeast^2^ ^,^ ^3^ ^,^ ^8^, North^6^, Midwest^7^, and South regions of the country^8^.

Two CxFV genotypes have been described^1^ ^,^ ^3^. Genotype 1 comprises sequences of CxFV detected in the USA and Asia. Genotype 2 comprises sequences of CxFV detected in the Caribbean, Latin America, and Africa. In the present study, we identified 982 bp sequences of the CxFV NS5 gene that grouped with sequences of CxFV previously detected in the Caribbean, Latin America, and Africa. Therefore, we conclude that the CxFV detected in Sao Paulo belongs to genotype 2. The viral isolates shared 94.89 to 100% similarity at the nucleotide level, considered high when compared to the similarity shared by all the sequences of CxFV included in the group of sequences from the Caribbean, Latin America, and Africa (88.48 - 100%). This finding indicates a low genetic diversity at the nucleotide level among CxFV present in mosquitoes in São Paulo, even though the sequences were detected in different species of mosquitoes and in parks located in different regions of the city.

Fernandes et al.^2^ screened mosquitoes from parks located in urban areas of São Paulo and reported the detection of both CxFV and Aedes flavivirus (AEFV). In their study, 74.8% of the mosquito pools analyzed consisted of *Culex* and 20.2% of *Aedes*. CxFV was detected in 2% and AEFV in 0.24% of the pools, respectively. In the present investigation, carried out with mosquitoes from different urban parks in São Paulo and collected one year later than those obtained in the study by Fernandes et al.^2^, the proportion of pools of *Aedes* mosquitoes analyzed was larger (45%). Therefore, we had anticipated finding AEFV in our samples. Surprisingly, only CxFV was detected in this study. This suggests that CxFV is a more widely disseminated ISF and that other ISFs encountered in *Aedes* mosquitoes, such as AEFV, might be less common.

The detection of CxFV in field-collected mosquitoes provides information on the occurrence, distribution, and frequency of this virus; however there is an urgent need to understand the role of CxFV. Therefore, it would be of great interest to determine whether a CxFV infection in cell lines and/or in mosquitoes affects the ability of a human-pathogenic flavivirus to replicate in the same host. If a CxFV infection interferes with the simultaneous infectivity, replication, or transmission of a pathogenic flavivirus, CxFV could be utilized to mitigate the transmission of medically important flaviviruses. Several studies with different ISFs have been performed to answer this question, but the results remain conflicting^14^ ^-^ ^15^.

In conclusion, genotype 2 of CxFV was detected in parks in the city of São Paulo. Its role in modulating the ability of pathogenic flaviviruses to infect mosquitoes and, therefore, to modulate the occurrence of epidemics should be a priority for future research.

## References

[1] Cook S, Moureau G, Harbach RE, Mukwaya L, Goodger K, Ssenfuka F (2009). Isolation of a novel species of flavivirus and a new strain of Culex flavivirus (Flaviviridae) from a natural mosquito population in Uganda. J Gen Virol.

[2] Fernandes LN, de Paula MB, Araújo AB, Gonçalves EF, Romano CM, Natal D (2016). Detection of Culex flavivirus and Aedes flavivirus nucleotide sequences in mosquitoes from parks in the city of São Paulo, Brazil. Acta Trop.

[3] Machado DC, Mondini A, Santana VS, Yonamine PTK, Chiaravalloti F, Zanotto PMA (2012). First Identification of Culex flavivirus (Flaviviridae) in Brazil. Intervirol.

[4] Hoshino K, Isawa H, Tsuda Y, Yano K, Sasaki T, Yuda M (2007). Genetic characterization of a new insect flavivirus isolated from Culex pipiens mosquito in Japan. Virol.

[5] Blitvich BJ, Firth AE (2015). Insect-specific flaviviruses: a systematic review of their discovery, host range, mode of transmission, superinfection exclusion potential and genomic organization. Viruses.

[6] Fernandes LN, Coletti TM, Monteiro FJC, Rego MODS, Ribeiro ESD, Ribeiro GO (2018). A Novel Highly Divergent Strain of Cell Fusing Agent Virus (CFAV) in Mosquitoes from the Brazilian Amazon Region. Viruses.

[7] Moraes OS, Cardoso BF, Pacheco TA, Pinto AZL, Carvalho MS, Hahn RC (2019). Natural infection by Culex flavivirus in Culex quinquefasciatus mosquitoes captured in Cuiabá, Mato Grosso Mid-Western Brazil. Med Vet Entomol.

[8] Gravina HD, Suzukawa AA, Zanluca C, Cardozo Segovia FM, Tschá MK, Martins da Silva A (2019). Identification of insect-specific flaviviruses in areas of Brazil and Paraguay experiencing endemic arbovirus transmission and the description of a novel flavivirus infecting Sabethes belisarioi. Virology.

[9] Goldani LZ (2017). Yellow fever outbreak in Brazil, 2017. Braz J Infect Dis.

[10] Paixão ES, Teixeira MG, Rodrigues LC (2018). Zika, chikungunya and dengue: the causes and threats of new and re-emerging arboviral diseases. BMJ Glob Health.

[11] Forattini OP (2002). Culicidologia Médica.

[12] Fulop L, Barrett ADT, Phillpotts R, Martin K, Leslie D, Titball RW (1993). Rapid identification of flaviviruses based on conserved NS5 gene sequences. Journal of virological Methods.

[13] Quick J, Grubaugh ND, Pullan ST, Claro IM, Smith AD, Gangavarapu K (2017). Multiplex PCR method for MinION and Illumina sequencing of Zika and other virus genomes directly from clinical samples. Nat Protoc.

[14] Zhang G, Asad S, Khromykh AA, Asgari S (2017). Cell fusing agent virus and dengue virus mutually interact in Aedes aegypti cell lines. Sci Rep.

[15] Baidaliuk A, Miot EF, Lequime S, Moltini-Conclois I, Delaigue F, Dabo S (2019). Cell-Fusing Agent Virus Reduces Arbovirus Dissemination in Aedes aegypti Mosquitoes In Vivo. J Virol.

